# Epidemiological insights into *Haemophilus influenzae* and *Pseudomonas aeruginosa* persistent colonization in non-cystic fibrosis bronchiectasis patients: a longitudinal and multicenter study

**DOI:** 10.1186/s12931-026-03553-1

**Published:** 2026-02-16

**Authors:** Irene Cadenas-Jiménez, Paula Camps-Massa, Filipe Gonçalves-Carvalho, Yasmina Benaali-Bakkar, Sara Quero, Laura Rodríguez, Adrián Antuori, Lucía Saiz-Escobedo, Sara Calvo-Silveria, Antonio Oliver, M Angeles Dominguez, Fe Tubau, Aida González-Díaz, Carmen Ardanuy, Salud Santos, Alicia Marin, Bronchiomics group, Elisenda Arque, Laura Millares, Marina Simon, Ignasi Garcia-Olivé, Cristina Prat-Aymerich, Alicia Lacoma, Esther Barreiro, Annie Navarro, Virginia Plasencia, Eva Cuchí, Javier Pomares, Concepción Montón, Antonio Casabella, Montserrat Vendrell, Gerard Muñoz, Eva Polverino, Antonia Llunell, Guillermo Suárez-Cuartin, Carmen Calero, Sara Martí

**Affiliations:** 1 Microbiology Department, Hospital Universitari Bellvitge, IDIBELL-UB, Barcelona, Spain; 2 Research Network for Respiratory Diseases (CIBERES), ISCIII, Madrid, Spain; 3 Department of Pathology and Experimental Therapeutics, University of Barcelona, Barcelona, Spain; 4 Pneumology Department, Hospital Universitari Bellvitge, IDIBELL-UB, Barcelona, Spain; 5 Respiratory Medicine Department, Hospital Germans Trias I Pujol, IGTP-UAB, Badalona, Spain; 6 Microbiology Department, Clinical Laboratory North Metropolitan Area, Hospital Germans Trias I Pujol, IGTP-UAB, Badalona, Spain; 7 Microbiology Department, Hospital Son Espases, IdISBa, Palma, Spain; 8 Research Network for Infectious Diseases (CIBERInfec), ISCIII, Madrid, Spain; 9 Department of Medicine, University of Barcelona, Barcelona, Spain

**Keywords:** Chronic infection, Non-CF bronchiectasis, Bacterial persistence, Antimicrobial resistance, Pathogen adaptation, Molecular epidemiology

## Abstract

**Background:**

Bronchiectasis is a chronic respiratory disease characterized by recurrent exacerbations and persistent inflammation, often associated with bacterial pathogens such as *Haemophilus influenzae* and *Pseudomonas aeruginosa*. Phenotypic adaptations (e.g., antimicrobial resistance) complicate treatment and worsen a patient’s quality of life.

**Methods:**

Between 2019 and 2020, we isolated 52 *H. influenzae* and 48 *P. aeruginosa* strains from 62 non-CF bronchiectasis patients across three scheduled visits and during exacerbation episodes. Antimicrobial susceptibility (assessed by microdilution) and phenotyping assays (motility and hypermutability) were performed. Whole genome sequencing was applied for analyses of resistance determinants, virulence factors, and genetic diversity.

**Results:**

Of the 62 patients, 31 were colonized by *H. influenzae*, 28 by *P. aeruginosa*, and 3 were co-colonized. Severe disease was predominantly linked to *P. aeruginosa* (70.6%), while exacerbations were more common with *H. influenzae* (81.8%). Multilocus sequence typing (MLST) revealed high genetic diversity, with ST1025 and ST253 most common in *H. influenzae* and *P. aeruginosa*, respectively. Antimicrobial resistance was low, but *H. influenzae* showed the highest resistance to cotrimoxazole (40.4%), while *P. aeruginosa* showed high resistance to aminoglycosides (27.1%) and fluoroquinolones (25%). Virulence profiling of *P. aeruginosa* identified 22.9% of strains as hypermutable, 27.1% as mucoid, 31.3% harboring the *exoU* gene, and 41.7% with impaired twitching motility. Persistent colonization occurred in 16 patients (25.8%), with antimicrobial resistance emerging following previous antimicrobial treatment in one case.

**Conclusions:**

In this cohort, *H. influenzae* and *P. aeruginosa* showed similar prevalence, high genetic diversity, and rare co-colonization. *P. aeruginosa* was associated with more severe disease, higher antimicrobial resistance, and hypermutability, whereas *H. influenzae* was associated with acute exacerbations.

**Supplementary Information:**

The online version contains supplementary material available at 10.1186/s12931-026-03553-1.

## Impact

This study enhances our understanding of non-cystic fibrosis bronchiectasis by delineating the distinct clinical impacts of *H. influenzae* and *P. aeruginosa* colonization. The strong association of *P. aeruginosa* with severe persistent disease, and *H. influenzae* with frequent exacerbation underscores the importance of precise microbiological diagnosis for tailored patient management. The high inter-patient genetic diversity of the bacterial isolates contrasts with the long-term clonal persistence observed within individual patients once colonization is established. This, combined with evidence of acquired resistance in both species, and the presence of key virulence factors in *P. aeruginosa* (such as hypermutability and mucoidy), highlights the complexity of pathogen adaptation to airways. Overall, these findings support a more personalized approach to bronchiectasis care, encouraging clinicians to incorporate pathogen-specific traits into treatment decisions. Additionally, this work establishes a foundation for future research targeting persistent colonization and virulence mechanisms to improve patient outcomes and quality of life.

## Background

Bronchiectasis is a chronic inflammatory lung disease characterized by the permanent widening of one or more bronchi [1]. It is considered a highly heterogeneous disease in terms of etiology, severity, and clinical outcomes [2]. Patients with bronchiectasis often have strong inflammatory responses to tissue damage, which can further compromise the airways. This dysfunction obstructs effective mucus clearance, promoting bacterial colonization of the lungs [3]. Chronic bronchial infection plays a crucial role in the pathophysiology of bronchiectasis, contributing to the cycle of inflammation, tissue damage, and impaired mucus clearance. This bacterial colonization is often caused by gram-negative microorganisms such as *Haemophilus influenzae* and *Pseudomonas aeruginosa,* which are the common gram negative bacteria isolated from the lungs of the bronchiectasis population, being both of them associated with poor clinical outcomes [4–6].

*H. influenzae* is a human restricted pathogen that forms part of the upper airway microbiota. It is found in 20%–50% of healthy children and 20%–30% of healthy adults [7]. The species comprises both encapsulated (a–f) and unencapsulated or nontypeable (NTHi) strains [8]*.* Since the introduction of a vaccine against *H. influenzae* serotype b*,* NTHi has become the most common cause of infection [9], being prevalent among patients with chronic obstructive pulmonary disease (COPD) [10] and bronchiectasis [11]. Chronic lung colonization by *H. influenzae* results in increased airway inflammation [12]. Despite the increasing recognition of *H. influenzae* as a public health concern, the molecular pathogenicity and adaptative mechanisms used by NTHi in bronchiectasis and other chronic respiratory diseases remain poorly understood.

By contrast, *P. aeruginosa* is a well-known opportunistic pathogen linked to a worse prognosis in patients with bronchiectasis [6]. Its colonization correlates with increased exacerbation frequency, hospital admissions, and mortality [13]. Unlike *H. influenzae*, the adaptative mechanisms of *P. aeruginosa* in chronic lung infection have been extensively reported, especially in patients with cystic fibrosis (CF) [14]. These adaptative changes include loss of motility, loss of type III secretion system proteins, loss of quorum sensing, conversion to a mucoid phenotype, reduced virulence, increased antibiotic resistance, and enhanced biofilm formation [15]. More broadly, *P. aeruginosa* plays a central role in chronic airway infections through the production of virulence factors such as elastases, exotoxin A, rhamnolipids, and pyocyanin, which damage airway epithelium, impair mucociliary clearance, and sustain neutrophilic inflammation [16, 17]. These processes contribute to airway remodeling and progressive structural lung damage, with colonization being often associated with accelerated lung function decline, poorer quality of life, and increased mortality in bronchiectasis cohorts [18, 19]. Despite that, few studies have addressed the persistence mechanisms of *P. aeruginosa* in the context of bronchiectasis [20, 21]. While both microorganisms are among the most frequently isolated pathogens in patients with bronchiectasis, they rarely coexist in the same individual. Recent evidence suggests that the microbiota of these patients is often dominated by either *P. aeruginosa* or *H. influenzae* [22]. Moreover, although both pathogens are linked to a poor prognosis, their effects on patient outcomes differ significantly. *P. aeruginosa* colonization is associated with more severe disease manifestations and worse outcomes, including lung function decline. By contrast, *H. influenzae* is often found in milder cases of bronchiectasis without necessarily impacting lung function or mortality [5].

Understanding the behavior and interrelation of *P. aeruginosa* and *H. influenzae* in bronchiectasis is crucial given their high prevalence and clinical significance. The presence of one pathogen over the other can significantly influence clinical outcomes and disease management. To date, however, no comprehensive studies have examined the phenotypic and genotypic characteristics of both *H. influenzae* and *P. aeruginosa* in non-CF bronchiectasis. Therefore, we performed a thorough characterization of these microorganisms in a population with non-CF bronchiectasis to provide a more complete understanding of how each pathogen adapts and impacts the disease.

## Methods

### Study design and strain selection

This longitudinal multicenter study comprised seven centers: Hospital Universitari Germans Trias i Pujol (HUGTiP), Mútua de Terrassa (MT), Hospital Universitari de Bellvitge (HUB), Hospital Corporació Sanitaria Parc Taulí (HCSPT), Hospital Universitari de Girona Dr. Josep Trueta (HUDJT), Consorci Sanitari de Terrassa (CST) and Hospital del Mar (HM).

Sputum samples from a total of 165 patients with non-CF bronchiectasis were collected during 2019–2020 at four visits: initial (V0), 6 months (V1), 12 months (V2), and during exacerbations (VE). Several microorganisms were isolated (Supplementary Table 1) and patients with at least one isolate of *H. influenzae* or *P. aeruginosa* were included in the study. Bronchiectasis severity was determined using the Bronchiectasis Severity Index (BSI), which was calculated for each patient following established parameters [23]. Patients were classified into mild (BSI score 0–4), moderate (BSI score 5–8), and severe (BSI score > 9) disease. *H. influenzae* was cultured on Chocolate agar (PolyViteX, BioMérieux) at 37 °C with 5% CO_2_, while *P. aeruginosa* was cultured on Columbia Blood agar (PolyViteX, BioMérieux) at 37 °C. Strain persistence was defined as those with the same sequence types (STs) isolated more than 3 months apart.

### Phenotypic characterization and antimicrobial susceptibility

Strains were routinely grown in Blood Agar (COS) plates and incubated at 37º overnight in the case of *P. aeruginosa.* For *H. influenzae*, strains were grown in Chocolate agar (PVX) plates and incubated 37º + 5% CO_2_ overnight. Strain identification was performed using MALDI-TOF (Bruker Biotyper). Mueller–Hinton agar was used for qualitative observation of colony morphology and phenotype, including pigment production and mucoid strains. Initial isolation and identification of bacterial strains were performed at each participating hospital laboratory, following their routine diagnostic protocols. All subsequent analyses were conducted at HUB, using the same procedures described below.

Antimicrobial susceptibility was tested by microdilution using commercial panels. For *P. aeruginosa,* MicroScan NM57 (Beckman Coulter) and Mueller–Hinton medium were used. For *H. influenzae*, STRHAE2 (Sensititre) and *Haemophilus* test medium (HTM) were used. Minimum inhibitory concentrations (MIC) were determined following European Committee on Antimicrobial Susceptibility Testing (EUCAST) [24] and Clinical and Laboratory Standards Institute (CLSI) criteria [25].

### Motility

Motility was studied in all *P. aeruginosa* strains as described previously [20]. Briefly, Luria Bertani (LB) plates were used with different agar concentrations to assess swarming (0.5% agar), swimming (0.3% agar), and twitching (1.5% agar). Plates were incubated for 48 h, and the growth area was measured by a single diameter reading. Experiments were performed in triplicate, with *P. aeruginosa* PAO1 as control.

### Hypermutability

Mutation frequencies were determined in *P. aeruginosa* strains by resistance to rifampicin, based on a previously described method [26]. Briefly, overnight cultures of each *P. aeruginosa* strain were grown in LB broth at 37 °C with shaking. Cultures were diluted 1:1000 and incubated in fresh LB broth overnight. Then, serial dilutions were plated on Mueller–Hinton agar with rifampicin (300 µg/mL) and without rifampicin. Strains were considered hypermutable when the mutation frequency was at least 20-fold higher than that obtained for control strain PAO1. All experiments were performed in triplicate, using *P. aeruginosa* PAO1 and a PAO1 MutS mutant as negative and positive controls, respectively. Mutation frequencies were calculated as the ratio of resistant colonies obtained for each isolate to the total number of colonies grown in absence of rifampicin.

### DNA extraction and WGS

All *P. aeruginosa* and *H. influenzae* strains were subject to whole genome sequencing (WGS). DNA was extracted using the QIAamp DNA Mini Kit (Qiagen) and quantified with a Qubit flex Fluorometer (Thermo Fisher Scientific). For *P. aeruginosa* strains, libraries were prepared using the DNA Prep Library Kit (Illumina) followed by paired-end sequencing (2 × 150) on a NextSeq platform (Illumina). For *H. influenzae* strains, libraries were prepared using the Nextera-XT DNA Library Preparation Kit (Illumina) followed by paired-end sequencing (2 × 300) on a MiSeq platform (Illumina). FastQ sequences were assembled with INNUca v4.2.0 pipeline (github.com/B-UMMI/INNUca), using default parameters. Briefy, a quality control of the reads was performed using FastQC (http://www.bioinformatics.babraham.ac.uk/projects/fastqc), followed by a read cleaning and trimming with Trimmomatic [27]. The genome was assembled using SPAdes [28] and was polished by Pilon [29]. FastQ sequences were deposited in the European Nucleotide Archive (ENA) with the project number PRJEB90145.

### Bioinformatic analysis

Multilocus sequence typing (MLST) was determined in all *H. influenzae* and *P. aeruginosa* genomes, using MLST software v2.4 (github.com/tseemann/mlst). Serotypes were determined in all *P. aeruginosa* genomes using PAst v1.0 from the Center for Genomic Epidemiology [30]. Screening of acquired resistance mechanisms and virulence factors in both species was assessed using the ResFinder [31] and VirulenceFinder [32] databases. In silico serotypes for *H. influenzae* were determined using hicap (github.com/scwatts/hicap); for *P. aeruginosa*, in silico serotypes were determined using PAst 1.0 from the Center for Genomic Epidemiology.

Additionally, screening of chromosomal mutations was assessed using Geneious R9 (version 9.1.7), with the *P. aeruginosa* PAO1 (NZ_CP129519.1) and *H. influenzae* Rd KW20 (L42023.1) sequences as reference. For phylogenetic analysis, the genome SNP alignment was obtained with Snippy’s core module (github.com/tseeman/Snippy) and subjected to the prediction and removal of recombinant regions using Gubbins software v2.3.1 [33]. Phylogenetic trees were constructed using *P. aeruginosa* PAO1 (NZ_CP129519.1) and *H. influenzae* HI375 (CP009610.1) as references. Phylogenetic tree visualization was performed using iTOL [34]. ICEs were also analysed with Geneious R9 (version 9.1.7) using the ICE*Hin1056* (AJ627386) and ICE*Hpa8f* (AM884335) sequences as references. Unknown genes were identified using BLASTn. The re­presentation of the ICEs was established using the Geneious R9 program and further refined with the information obtained from BLASTn analysis using Inkscape.

## Results

### Bacterial epidemiology

**Fig. 1:**
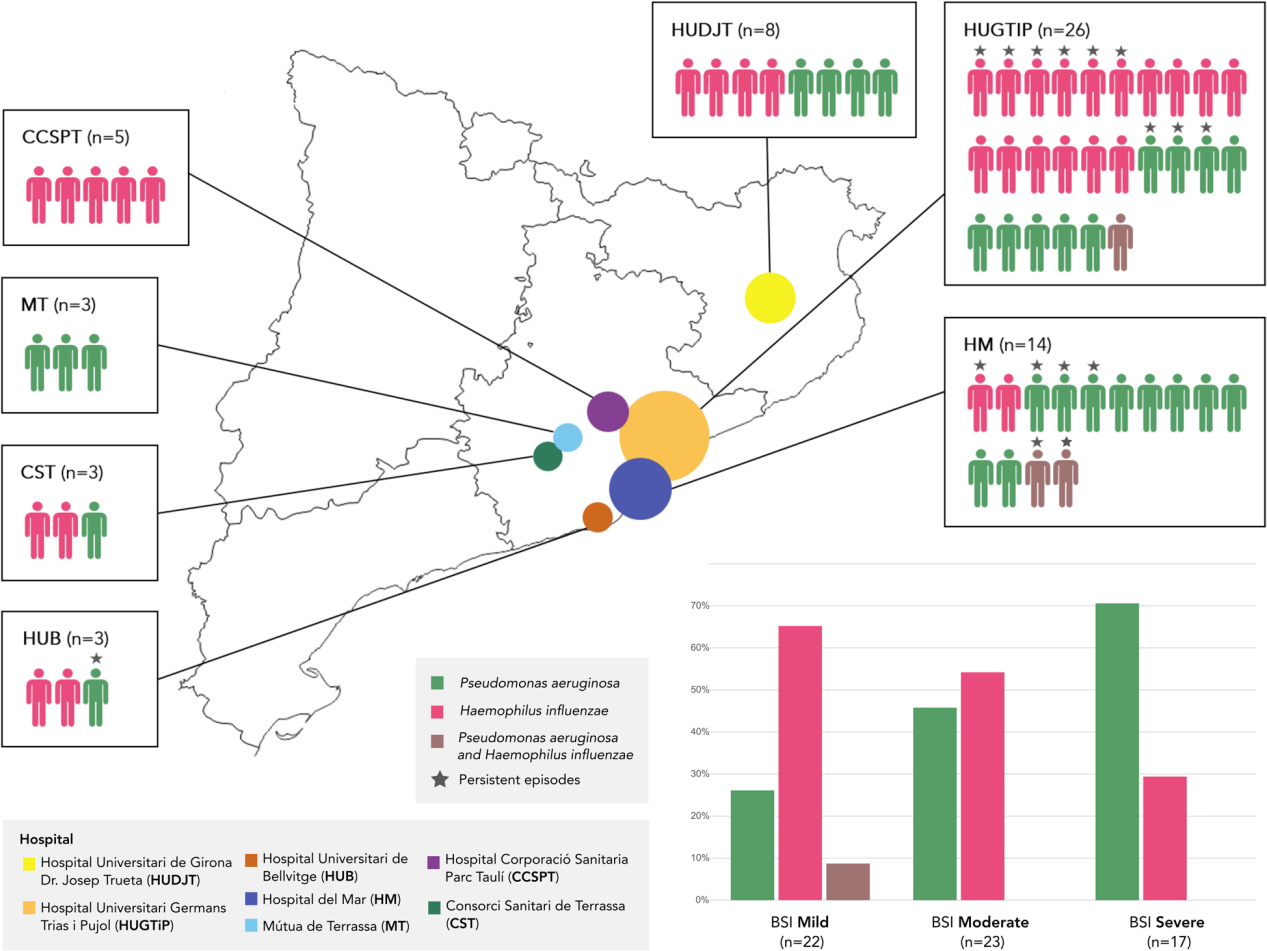
Distribution of patients and isolated species from different healthcare centers in Catalonia. Patients with *P. aeruginosa* are represented in green, patients with *H. influenzae* are represented in pink, and patients with both microorganisms are in brown. Stars represent patients with persistent episodes. The bar graph shows the percentage of each isolated species, stratified by Bronchiectasis Severity Index (BSI) (mild, moderate and severe)

A total of 52 *H. influenzae* and 48 *P. aeruginosa* strains were isolated from 62 patients at seven Spanish hospitals during the period from 2019 to 2020. The number of strains per patient ranged from one to five. Among the centers, HUGTiP followed by HM accumulated the highest percentages of patients and bacterial strains. CCSPT only included patients with *H. influenzae* strains, while MT only included patients with *P. aeruginosa* strains. Overall, 31 patients had *H. influenzae* strains, 28 had *P. aeruginosa* strains, and three were co-colonized. Analysis using the BSI revealed a predominance of moderate disease (*n =* 23), closely followed by mild disease (*n =* 22), and severe disease (*n =* 17); of note, exacerbations occurred in 11 patients. Among patients with severe disease, 70.6% (*n =* 12) were colonized by *P. aeruginosa*, whereas among patients with mild disease 68.2% (*n =* 17) were colonized by *H. influenzae* (Fig. 1). Moreover, exacerbations were not linked to a worse disease state, being more frequently associated with *H. influenzae* (*n =* 9; 81.8%) than *P. aeruginosa* (*n =* 2; 18.2%).

**Fig. 2:**
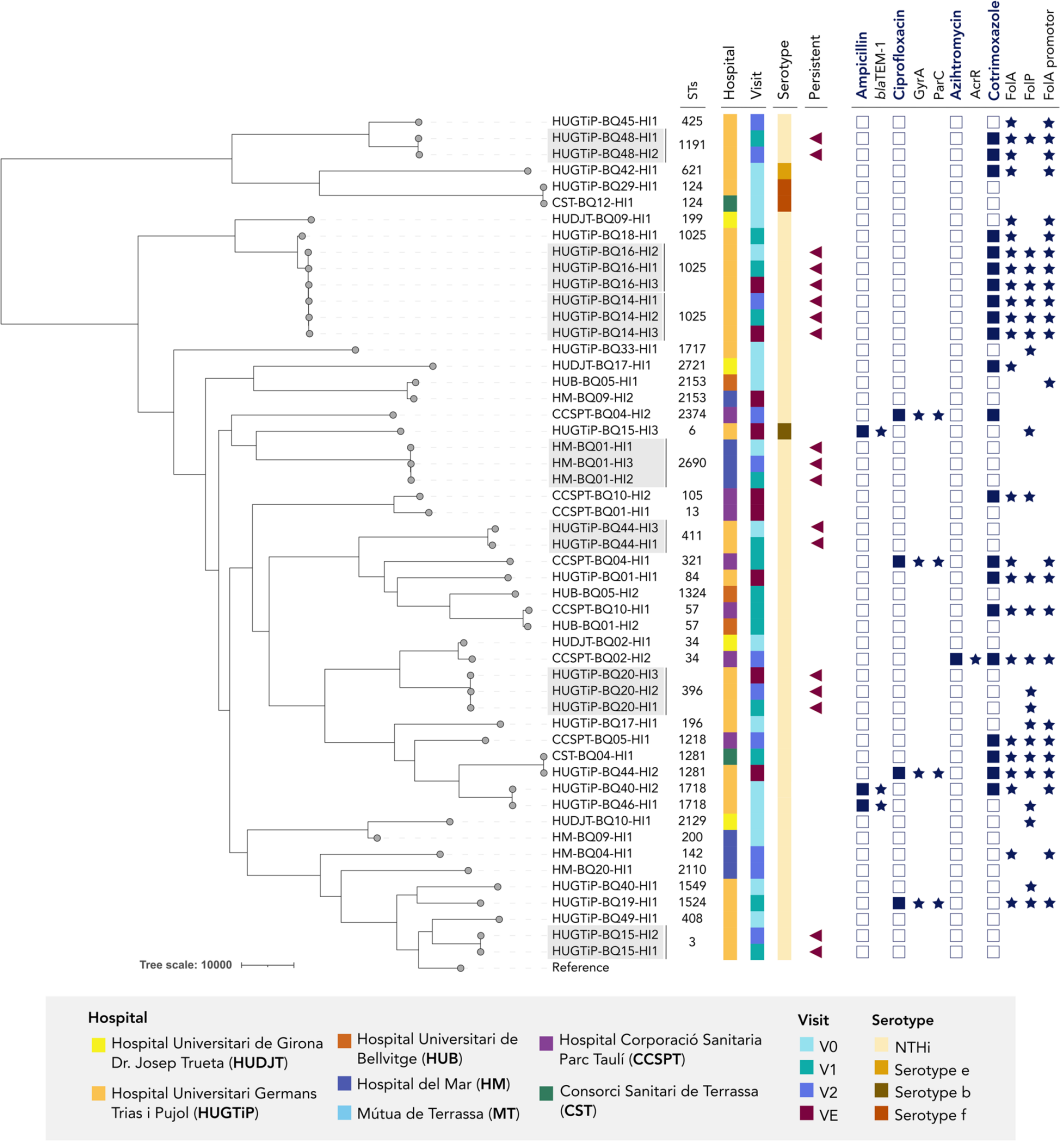
Phylogenetic tree of all *H. influenzae* isolates included in the study. Strain metadata includes multilocus sequence type (MLST), serotype, patient information (hospital and visit), and persistence status (persistent strains highlighted in grey). Antimicrobial resistance profiles are represented by filled squares. Specific amino acid substitutions linked to resistance are marked with stars. *v0: initial visit, v1: 6 months, v2: 12 months, vE: exacerbation visit

**Fig. 3:**
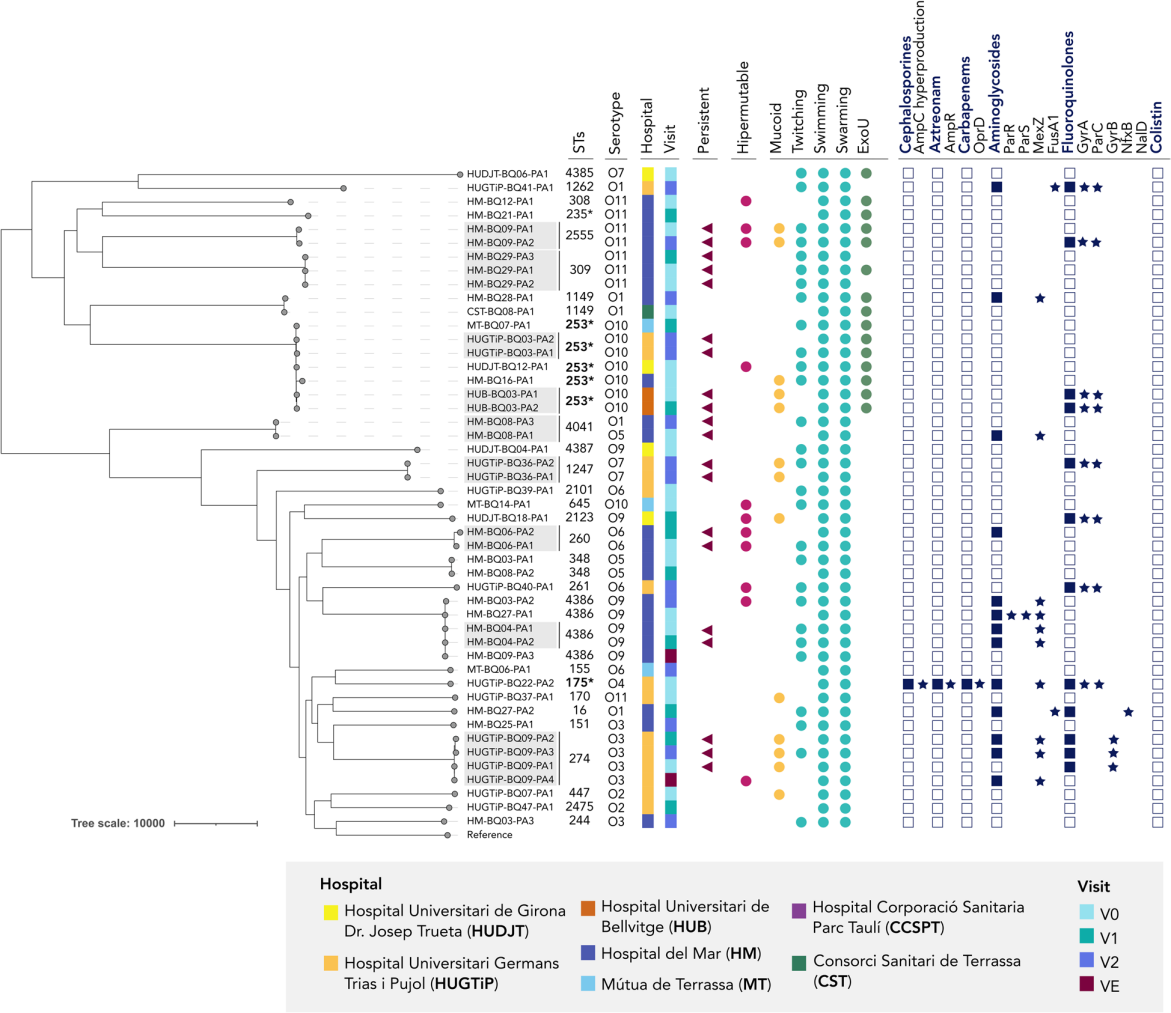
Phylogenetic tree of all *P. aeruginosa* isolates included in the study. Strain metadata includes multilocus sequence type (MLST), serotype, patient information (hospital and visit), and persistence status (persistent strains highlighted in grey). Virulence phenotypes, including hypermutability, mucoid morphology, motility (twitching, swimming, and swarming), and presence of the *exoU* gene, are indicated by filled circles. Antimicrobial resistance profiles are represented by filled squares. Specific amino acid substitutions linked to resistance are marked with stars.. *V0: initial visit, v1: 6 months, v2: 12 months, vE: exacerbation visit

MLST revealed significant genetic diversity for both species (Supplementary Fig. 1). For *H. influenzae,* 33 distinct STs were identified. ST1025 was the most common, found in 7 strains from three patients, all coming from HUGTiP. Non-typeable strains showed high diversity, with 30 unique STs. We detected four capsulated strains: one serotype b (ST6), one serotype e (ST621), and two serotype f (ST124) (Fig. 2). For *P. aeruginosa*, 27 different STs were found. The most prevalent was the high-risk clone ST253 (*n =* 7), isolated from five patients in five different hospitals. ST4386 was also common, associated with aminoglycoside resistance, and isolated in only 4 patients from HM. The high-risk clones ST175 and ST235 were underrepresented in our bronchiectasis population, with only one strain of each detected (Fig. 3). Serotypes O11 and O10 were the most frequent, with serotype O10 related to the high-risk clone ST253.

### Antimicrobial resistance

#### *Haemophilus influenzae*

The *H. influenzae* strains from patients with bronchiectasis showed high antibiotic susceptibility. Non-susceptible strains were resistant to cotrimoxazole (*n =* 21; 40.4%), fluoroquinolones (*n =* 4; 7.7%), ampicillin (*n =* 3; 5.8%), and azithromycin (*n =* 1; 1.9%) (Table 1).

**Table 1:**
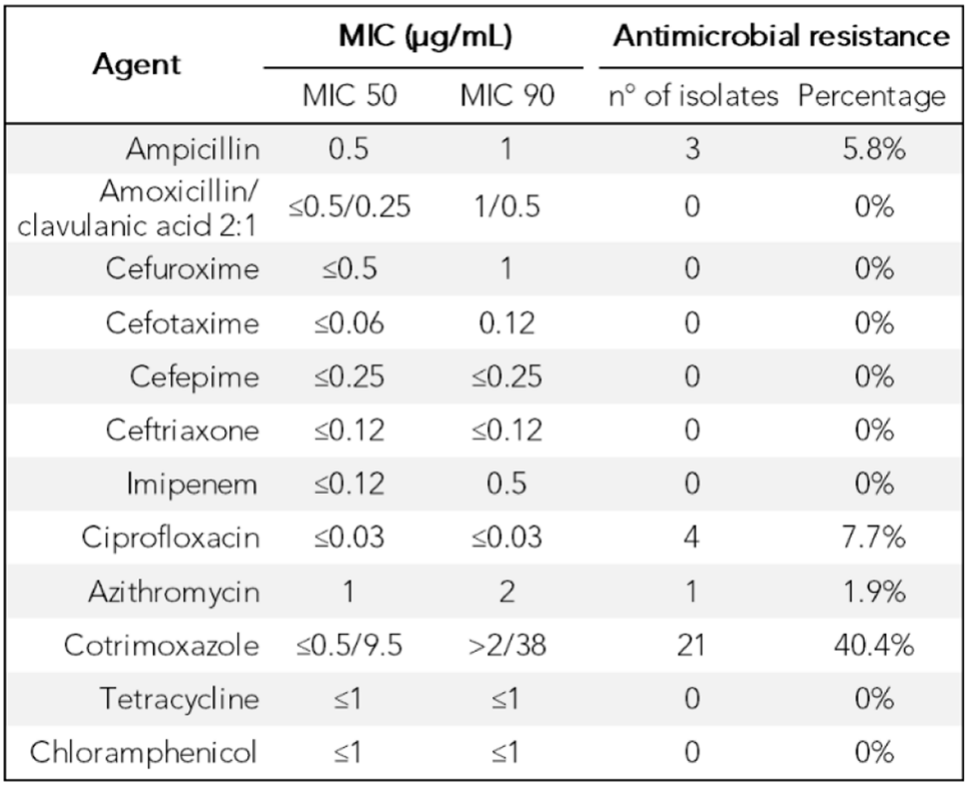
Antimicrobial resistance and minimum inhibitory concentrations (MIC) of* Haemophilus influenzae*

Antimicrobial resistance was mostly due to chromosomal point mutations. Cotrimoxazole resistance involved modifications in the FolA (I95L as most relevant), FolP (P64E) and FolA promoter regions (Supplementary Table 2). β-lactam resistance was attributed to *bla*_TEM-1_ acquisition in three strains. Although 29 strains had modifications in the PBP3 protein that were insufficient to confer resistance, group II strains had reduced susceptibility to ampicillin (Supplementary Table 3). Ciprofloxacin-resistant strains had modifications in the quinolone resistance determining region of GyrA (S84L and D88G) and ParC (S84I). The only azithromycin-resistant strain had an insertion in the negative regulator *acrR,* resulting in a premature stop codon.

**Fig. 4:**
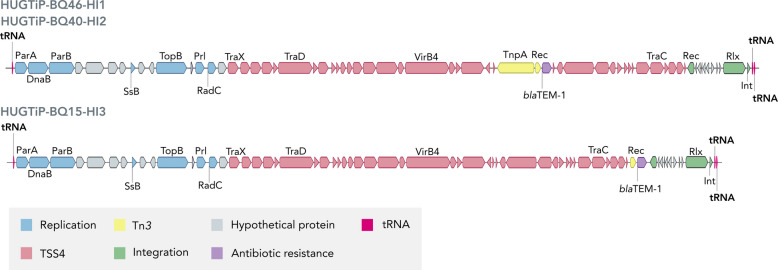
Integrative and conjugative elements found in *H. influenzae* strains. ICE*Hpa8f* was found in strain HUGTiP-BQ46-HI1 while a novel ICE called ICE*Hpa8f-like* was observed in strain HUGTiP-BQ15-HI3. In both ICEs the beta-lactamase *bla*TEM-1 is located in the Tn3. The structure of ICE*Hpa8f* in strain HUGTiP-BQ46-HI1 was conserved, while ICE*Hpa8f-like* had some structural differences. Transposon Tn3 is located between type 4 secretion system genes and recombination genes. Furthermore, the Tn3 consisted only in the recombinase and the beta-lactamase *bla*TEM-1 but is lacking the transposase TnpA

The analysis of mobile genetic elements was focused on strains harboring the *bla*_TEM-1_ β-lactamase gene. In all cases, this gene was located within an integrative and conjugative element (ICE). Specifically, the known element ICE*Hpa8f* was identified in strain HUGTiP-BQ46-HI1, while a novel variant, designated ICE*Hpa8f-like*, was found in strain HUGTiP-BQ15-HI3. In both cases, the *bla*_TEM-1_ was embedded within a Tn3 transposon. However, the genetic context of the transposon differed between the two ICEs. In ICE*Hpa8f*, the Tn3 (containing the *bla*_TEM-1_, a recombinase and the transposase TnpA) was inserted directly within the genes encoding the type IV secretion system. In contrast, the Tn3 transposon in the ICE*Hpa8f-like* was found at a different insertion site, located between the type IV secretion system genes and recombination genes. Furthermore, this Tn3 variant lacked the *tnpA* gene, containing only the *bla*_TEM-1_ and a recombinase (Fig. 4).

### *Pseudomonas aeruginosa*

Antimicrobial susceptibility patterns for *P. aeruginosa* are shown in Table 2. The highest resistance rates were observed for aminoglycosides (*n =* 13; 27.1%) and ciprofloxacin (*n =* 12; 25%). β-lactams had the lowest resistance rates, with only one strain (ST175 high-risk clone) resistant to penicillins, cephalosporins, monobactams, and carbapenems.

**Table 2:**
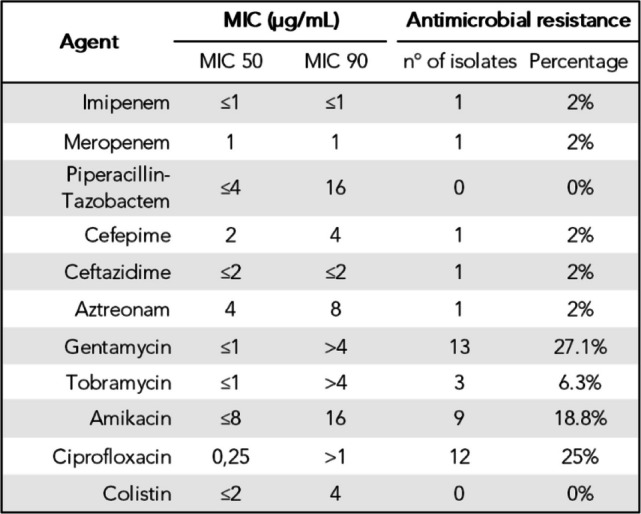
Antimicrobial resistance and minimum inhibitory concentrations (MIC) of *Pseudomonas aeruginosa*

In all cases, antimicrobial resistance was likely associated with the acquisition of chromosomal point mutations (Fig. 3). Aminoglycoside resistance was mostly produced by MexXY-OprM efflux pump overexpression due to modifications in the regulator MexZ (*n =* 10), followed by modifications in FusA1 (*n =* 2) and by MexEF-OprN overexpression due to modifications in ParR/S (*n =* 1). Ciprofloxacin resistance predominantly involved DNA gyrase and topoisomerase IV modifications, with GyrA and ParC modifications being the most common (*n =* 8), followed by GyrB (*n =* 3). One strain was ciprofloxacin-resistant due to MexCD-OprJ efflux pump overexpression via NfxB modifications (Supplementary Dataset 1). Resistance to piperacillin and cephalosporines was attributed to modifications in the AmpR regulator (G154R and G273E). Imipenem resistance was linked to a truncated OprD porine, and resistance to meropenem was explained by MexAB-OprM efflux pump overexpression.

### Persistence

**Fig. 5:**
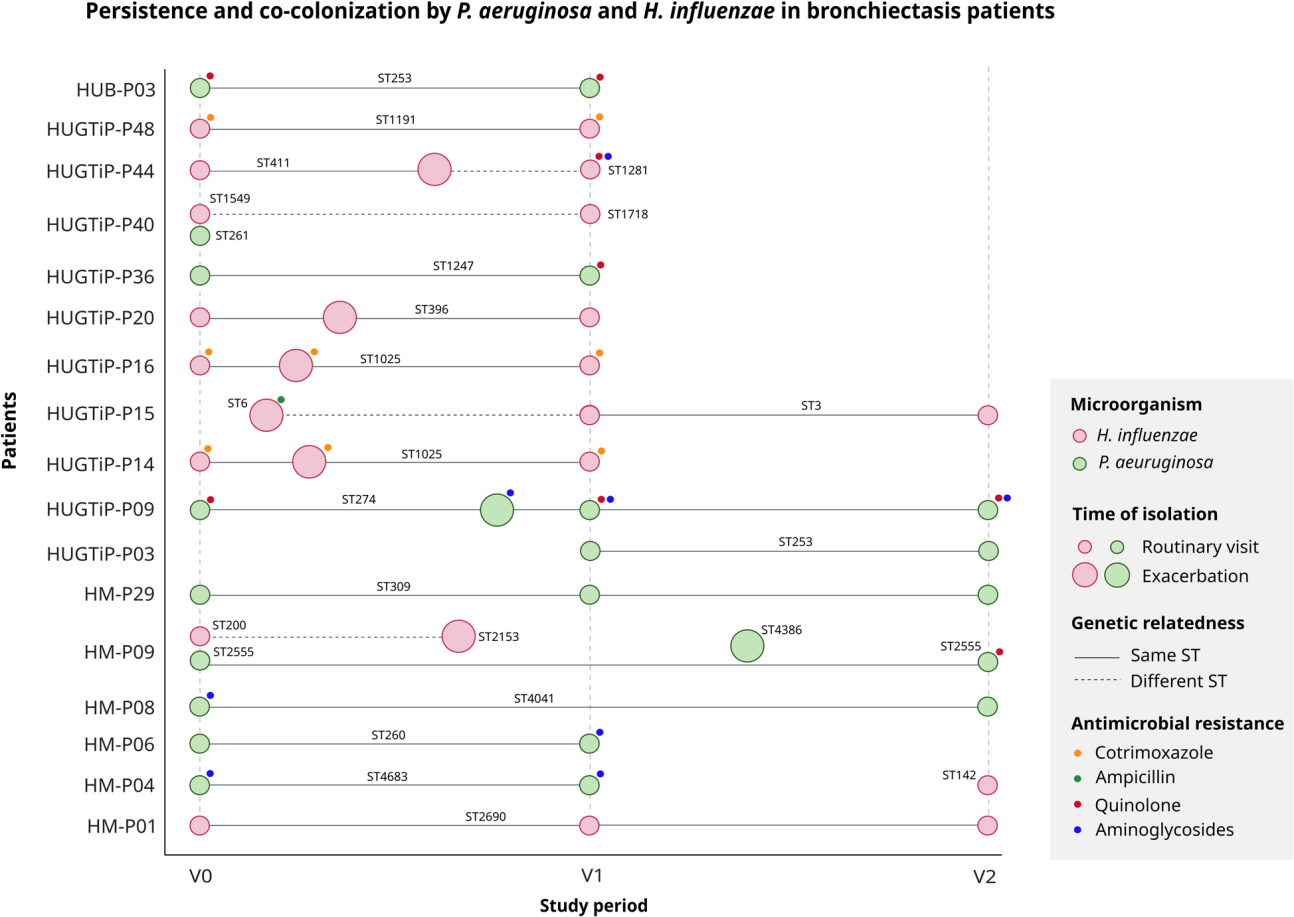
Temporal dynamics of persistent colonization and resistance evolution. Longitudinal overview of persistent bacterial colonization by *H. influenzae* (pink) and *P. aeruginosa* (green) isolates, including episodes of patient co-colonization. The figure displays the temporal sequence of isolate collection, genetic relatedness between sequential isolates, and evolution of antimicrobial resistance patterns throughout the study period

Strain persistence was not frequent in our cohort. MLST analysis indicated that 16 patients were colonized by *H. influenzae* or *P. aeruginosa* for at least three months (25.8%). No significant differences between species were observed: nine patients (57.1%) were colonized persistently by *P. aeruginosa* and seven patients (42.9%) by *H. influenzae* (Fig. 5).

Overall, 6 patients with chronic *H. influenzae* colonization and 2 patients with persistent *P. aeruginosa* colonization experienced acute exacerbations during the study period. Moreover, five patients acquired antimicrobial resistance, which was more common with *P. aeruginosa* (*n =* 4; 75%) than *H. influenzae* (*n =* 1; 25%). The persistent *H. influenzae* strain acquired quinolone and cotrimoxazole resistance by acquiring modifications in GyrA/ParC and FolA/FolP, respectively. The persistent *P. aeruginosa* strains acquired resistance to aminoglycosides and quinolones via modifications in NalD and GryA/ParC, respectively. Among the five patients harboring persistent strains that acquired antimicrobial resistance, two cases developed following acute exacerbations. For patient HM-P09, moxifloxacin was successfully used to treat an exacerbation caused by a sporadic *P. aeruginosa* clone (ST4386) but this led to fluoroquinolone resistance in the persistent *P. aeruginosa* clone (ST2555).

### Hypermutability and virulence in *P. aeruginosa*

In total, 11 hypermutable strains were identified from nine patients (22.9%). The median mutation frequency for the hypermutator strains was 4.44 × 10⁻⁶.This phenotype was mostly explained by modifications in genes involved in the mismatch repair system. Most strains harbored amino acid modifications in MutL (*n =* 7), followed by UvrD, SodM, or Ung. Notably, in patients carrying persistent strains that had also developed a hypermutable phenotype, this elevated mutation rate was implicated in the acquisition of antimicrobial resistance during the study period (Fig. 3). This is exemplified by patient HM-P06, who developed aminoglycoside resistance over time, and patient HM-P09, who developed fluoroquinolone resistance by visit 2 (Fig. 5).

Besides hypermutability, the most frequent virulence mechanism was alginate overproduction (*n =* 13; 27.1%), caused mainly by mutations in the *mucA* gene (*n =* 7). It was noted that six strains had *mucA* mutations without expressing a mucoid phenotype, suggesting a reversion process: the mucoid revertant phenotype could be explained by modifications in AlgU in three of these strains, but the mechanism remained unidentified in the other three strains.

The exotoxin ExoU was also prevalent among the *P. aeruginosa* collection, with 15 strains presenting an ExoU + phenotype (31.3%). Phylogenetic analysis showed that ExoU + and ExoS + strains formed two different clusters (Fig. 3). The presence of the *exoU* gene was related to the high-risk clones ST235 and ST253. Finally, regarding motility, all strains demonstrated the capability for both swimming and swarming, but only 58.3% had twitching motility.

## Discussion

Bronchiectasis is among the most prevalent chronic respiratory diseases, with *H. influenzae* and *P. aeruginosa* being the most common microorganisms responsible for exacerbations and chronic colonization [6, 16]. These opportunistic pathogens are continuously exposed to antibiotic and immune system pressures that can lead to genetic and phenotypic adaptations to survive this hostile environment. This study provides a comprehensive molecular characterization of *H. influenzae* and *P. aeruginosa* in a population of non-CF bronchiectasis patients.

Both microorganisms had a similar prevalence. Consistent with previous research, *P. aeruginosa* correlated with severe disease, based on BSI scores [19], while *H. influenzae* dominated in milder and early stages of chronic respiratory diseases [5]. This aligns with the proposed disease progression model in COPD, where *H. influenzae* colonization often precedes *P. aeruginosa* isolation [35], suggesting that *H. influenzae* colonization may predispose individuals to subsequent *P. aeruginosa* infection, potentially explaining the pathogen-specific severity gradient. The association of *P. aeruginosa* with greater disease severity could be explained due to its capacity to sustain chronic infection and drive progressive lung damage. Biofilm formation and the presence of mucoid strains promote persistent inflammation and structural airway injury, while hypermutable strains accelerate resistance acquisition, limiting treatment options. These mechanisms translate clinically into faster lung function decline, and increased mortality [36, 37].

Of note, our data showed that *H. influenzae* was the primary cause of exacerbations, contrasting with previous studies where both pathogens were present at similar percentages [21, 38]. Although colonization with *P. aeruginosa* has been identified as an independent risk factor for recurrent exacerbations [39, 40], the relatively short one-year observation period may have been insufficient to capture the increase in exacerbations typically associated with *P. aeruginosa* colonization. Despite the exacerbation status, patients colonized with *P. aeruginosa* had significantly higher disease severity, suggesting that, while exacerbations are an important component of the BSI [23], other factors such as chronic inflammation or lung damage may play a critical role in driving disease severity. Nevertheless, persistent colonization with *H. influenzae* or *P. aeruginosa* was found in 16 patients, where the same bacterial clone was consistently isolated across all sampled time points, including periods of clinical stability and exacerbation. This finding is consistent with several microbiome studies showing stable microbial communities between stable and exacerbation phases [41–43]. This underscores the importance of host–microbe adaptation, with shifts in the composition of existing lung microbes rather than the introduction of a new pathogen.

The study revealed significant clonal diversity among *H. influenzae* strains, with 33 distinct ST identified. The most common was ST1025, detected in three patients, two of whom had long-term colonization, suggesting potential for persistence. Although ST1025 has not been previously reported in patients with bronchiectasis and has not been associated with persistence, it has been linked to COPD [44], indicating a potential role in chronic respiratory diseases. Additionally, ST3 and ST57, which have been previously reported as two of the most prevalent *H. influenzae* lineages [9], were also found in one and two patients, respectively. This study confirmed the high prevalence of NTHi [45], although one serotype b strain, belonging to the already documented ST6, was linked to an acute exacerbation, underscoring the importance of ongoing surveillance. Among other capsulated strains, serotype f belonged to the well-documented ST124 clone [46], and serotype e was identified as ST621, a single locus variant of ST18 and ST122 associated with this serotype [47]. Antimicrobial resistance in *H. influenzae* remained low, except for cotrimoxazole, consistent with previous reports [45]. β-lactam resistance was primarily mediated by a TEM-1 β-lactamase, located on two related ICEs: the previously described ICE*Hpa8f* [48] and a novel ICE*Hpa8f-like* variant. Notably, all acquired resistance genes in this study were located within ICEs, reinforcing their established role as primary vectors of antimicrobial resistance acquisition in *H. influenzae,* as seen previously in azithromycin-resistant isolates [49]. The identification of ICE*Hpa8f* variants in both *H. influenzae* and *H. parainfluenzae* strains [48–50], underscores the potential for interspecies dissemination and highlights the need for surveillance of these mobile genetic elements. Fluoroquinolone resistance, while still low (7.4%), showed a slight increase compared to earlier studies, consistent with recent trends observed in invasive *H. influenzae* strains [51].

The clonal distribution of *P. aeruginosa* differed from the general trends in Spain, where globally prevalent high-risk clones such as ST175 and ST235 typically predominate [52]. By contrast, each of these clones was found in only one case, while ST253, usually considered less common, emerged as the most common strain. This divergence from national patterns is consistent with previous observations showing that the clonal distribution of *P. aeruginosa* in bronchiectasis often differs from that seen in other diseases [53]. The predominance of ST253, a clone more closely associated with virulence rather than resistance, along with the low representation of multidrug and extensively drug resistant clones, may explain the lower antibiotic resistance levels in our cohort compared to larger *P. aeruginosa* cohorts [54, 55]. These findings support earlier longitudinal bronchiectasis studies [21] and recent surveys indicating a decline in *P. aeruginosa* resistance [56].

Antimicrobial resistance in *P. aeruginosa* was explained by the acquisition of chromosomal point mutations. Regarding aminoglycosides, MexXY-OprM efflux pump overexpression was the most common mechanism, mainly caused by modifications in MexZ, but we observed no acquisition of aminoglycoside modifying enzymes. Modifications in MexZ are more commonly seen in advanced CF, suggesting a positive selection of these modifications during the establishment of chronic colonization [57]. Resistance to cephalosporines was associated with chromosomal β-lactamase AmpC overexpression [58], while carbapenem resistance was associated to the presence of a truncated OprD porin [54, 59] and to mutations that leads to the overexpression of MexAB-OprM [58]. Interestingly, no acquired extended spectrum β-lactamases were detected in the cohort, again probably reflecting the absence of high-risk clones typically linked to these resistance determinants [59].

A high proportion of hypermutable strains was identified, a trait comparable to those observed in CF [26]. This may suggest that chronic airway colonization in non-CF bronchiectasis may also promote genetic adaptations that increase mutation rates, potentially in response to long-term antibiotic exposure or host immune pressure. Moreover, phenotypes associated with persistent infection in CF, such as mucoid morphology and reduced twitching motility, were frequently observed [60], highlighting a convergent adaptive response to the lung environment. These phenotypic traits have important clinical consequences. Hypermutable strains, frequently reported in chronic airway infections, accelerate the emergence of multidrug resistance and may contribute to treatment failure [26]. Regarding mucoid phenotype, driven by alginate overproduction, it enhances biofilm stability and protects bacteria against phagocytosis, thereby promoting immune evasion and long-term persistence [61]. Finally, loss of twitching motility, mediated by type IV pili, may reduce bacterial dispersal but favors localized biofilm maturation, reinforcing chronic colonization. Together, these adaptations illustrate how *P. aeruginosa* evolves to persist in the bronchiectatic lung, with direct implications in the risk of exacerbations and lung function decline [18].

The prevalence of ExoU-positive strains in our cohort (31.3%) is consistent with previous reports from Spain regarding clinical *P. aeruginosa* isolates [54]. Although the presence of ExoU has been correlated with unfavorable outcomes in clinical and experimental research [62], we found no association between the presence of ExoU and BSI categories.

A major strength of this study is its multicenter design, which includes data from seven Spanish hospitals.This greatly enhances the diversity of our patient population and increases the applicability of our findings across different clinical settings. However, some limitations must be considered. First, the overall cohort size, through multicentric, remains moderate and may limit the generalizability of the findings to broader populations. Second, the one-year follow-up period, while providing robust initial data, may be too short to analyze the long-term trends in microbial colonization and the evolution of chronic infection. Finally, our study did not include formal statistical testing due to the small sample size, which limits the strength of the comparisons.

## Conclusion

In conclusion, this study provides a microbiological characterization of *H. influenzae* and *P. aeruginosa* in patients with non-CF bronchiectasis. *P. aeruginosa* was associated with more severe disease and *H. influenzae* was more frequently associated with exacerbations. Both pathogens showed significant genetic diversity, and co-colonization was uncommon. The clonal distribution of *P. aeruginosa* differed from that reported elsewhere in Spain. The notable presence of hypermutable strains and a mucoid phenotype in *P. aeruginosa* suggests adaptive mechanisms that facilitate chronic infection.

## Supplementary Information

Supplementary Material 1: Supplementary Figure 1. Phylogenetic trees showing different STs found among *H. influenzae* (A) and *P. aeruginosa* (B) strains regarding hospitals. Supplementary Table 1. Microorganisms isolated from the 165 non-CF bronchiectasis patients included in the study (2019-2020). *High quality sputum samples were considered for the study. Supplementary Table 2. Cotrimoxazole susceptibility results, tested by microdilution in *Haemophilus influenzae* strains grown over Mueller–Hinton-Fastidious medium (EUCAST criteria), and associated amino acid modifications in FolA and FolP and mutations in the *folA* promotor. Supplementary Table 3. Amino acid substitutions identified in the penicillin-binding protein 3 of *H. influenzae* strains.

Supplementary Material 2.

## Data Availability

The authors confirm that all supporting data and protocols have been provided within the article or through supplementary data files.

## Abbreviations

NTHi: Nontypeable Haemophilus influenzae
COPD: Chronic Obstructive Pulmonary Disease
CF: Cystic Fibrosis
HUGTiP: Hospital Universitari Germans Trias i Pujol
MT: Mutua de Terrassa
HUB: Hospital Universitari de Bellvitge
HCSPT: Hospital Corporació Sanitaria Parc Taulí
HUDJT: Hospital Universitari de Girona Dr. Josep Trueta
CST: Consorci Sanitari de Terrassa
HM: Hospital del Mar
BSI: Bronchiectasis severity index
MLST: Multilocus sequence typing
ST: Sequence types
COS: Blood Agar
PVX: Chocolate agar
MH: Mueller–Hinton
HTM: *Haemophilus* Test medium
MIC: Minimum inhibitory concentrations
EUCAST: European Committee on Antimicrobial Susceptibility Testing
LB: Luria Bertani
WGS: Whole genome sequencing
ICE: Integrative and conjugative element
