# Supplementary figures and images for: Epidemiological insights into Haemophilus influenzae and Pseudomonas aeruginosa persistent colonization in non-cystic fibrosis bronchiectasis patients: a longitudinal and multicenter study
Source: Respir Res. 2026 Feb 16;27:87. doi: 10.1186/s12931-026-03553-1 (PMC12918164; doi:10.1186/s12931-026-03553-1)

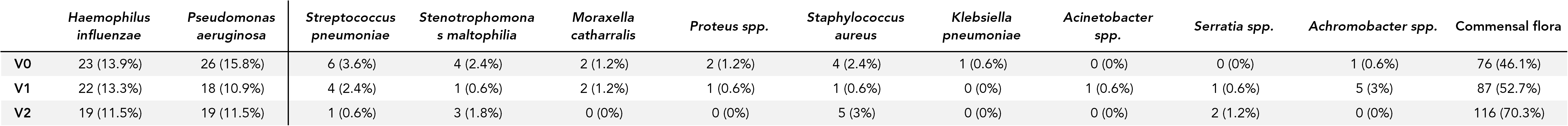

Supplement: Supplementary file 1 — Supplementary Material 1: Supplementary Figure 1. Phylogenetic trees showing different STs found among H. influenzae (A) and P. aeruginosa (B) strains regarding hospitals. Supplementary Table 1. Microorganisms isolated from the 165 non-CF bronchiectasis patients included in the study (2019-2020). *High quality sputum samples were considered for the study. Supplementary Table 2. Cotrimoxazole susceptibility results, tested by microdilution in Haemophilus influenzae strains grown over Mueller–Hinton-Fastidious medium (EUCAST criteria), and associated amino acid modifications in FolA and FolP and mutations in the folA promotor. Supplementary Table 3. Amino acid substitutions identified in the penicillin-binding protein 3 of H. influenzae strains. [file 12931_2026_3553_MOESM1_ESM.zip › Supplementary Table 1.png]

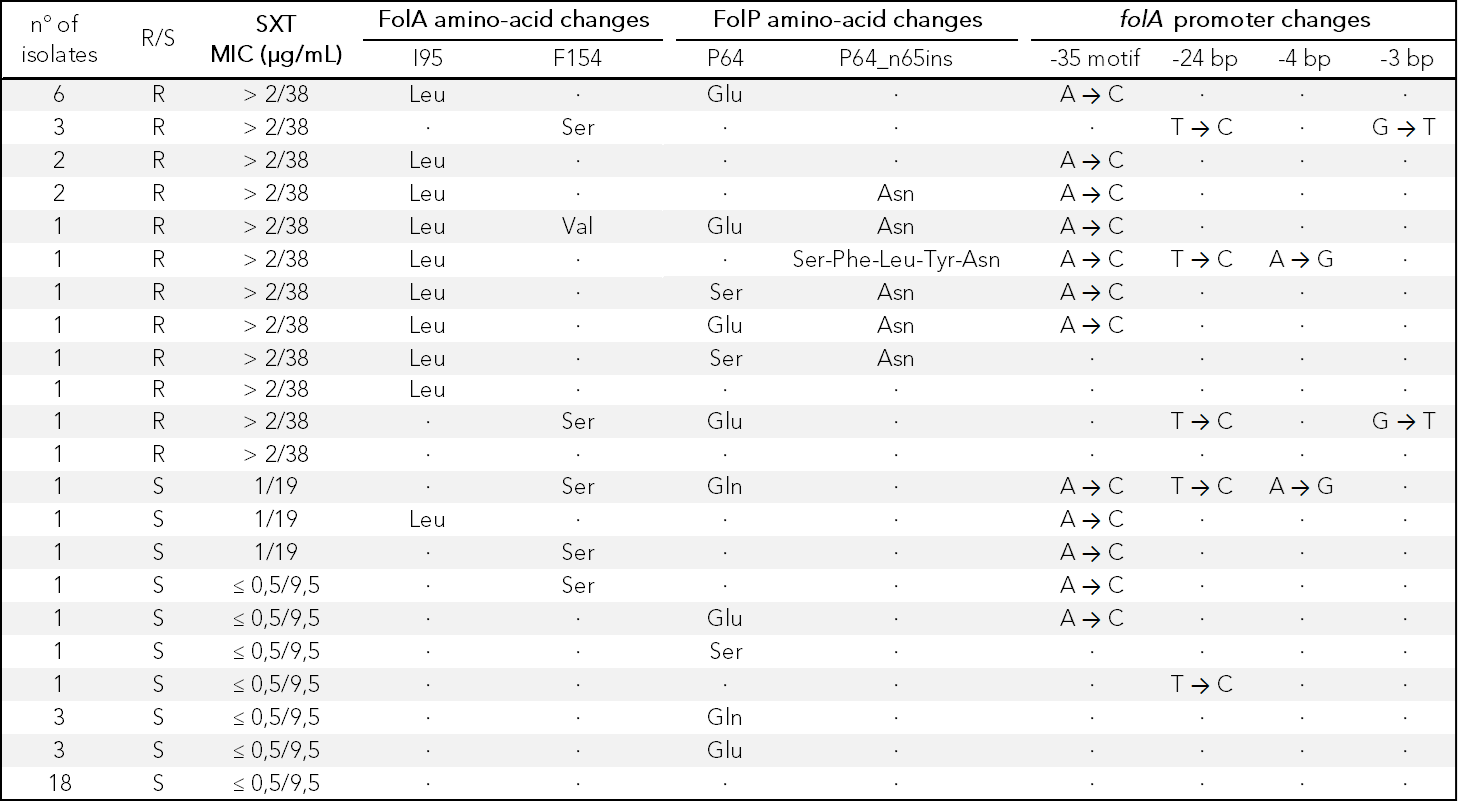

Supplement: Supplementary file 1 — Supplementary Material 1: Supplementary Figure 1. Phylogenetic trees showing different STs found among H. influenzae (A) and P. aeruginosa (B) strains regarding hospitals. Supplementary Table 1. Microorganisms isolated from the 165 non-CF bronchiectasis patients included in the study (2019-2020). *High quality sputum samples were considered for the study. Supplementary Table 2. Cotrimoxazole susceptibility results, tested by microdilution in Haemophilus influenzae strains grown over Mueller–Hinton-Fastidious medium (EUCAST criteria), and associated amino acid modifications in FolA and FolP and mutations in the folA promotor. Supplementary Table 3. Amino acid substitutions identified in the penicillin-binding protein 3 of H. influenzae strains. [file 12931_2026_3553_MOESM1_ESM.zip › Supplementary Table 2.png]

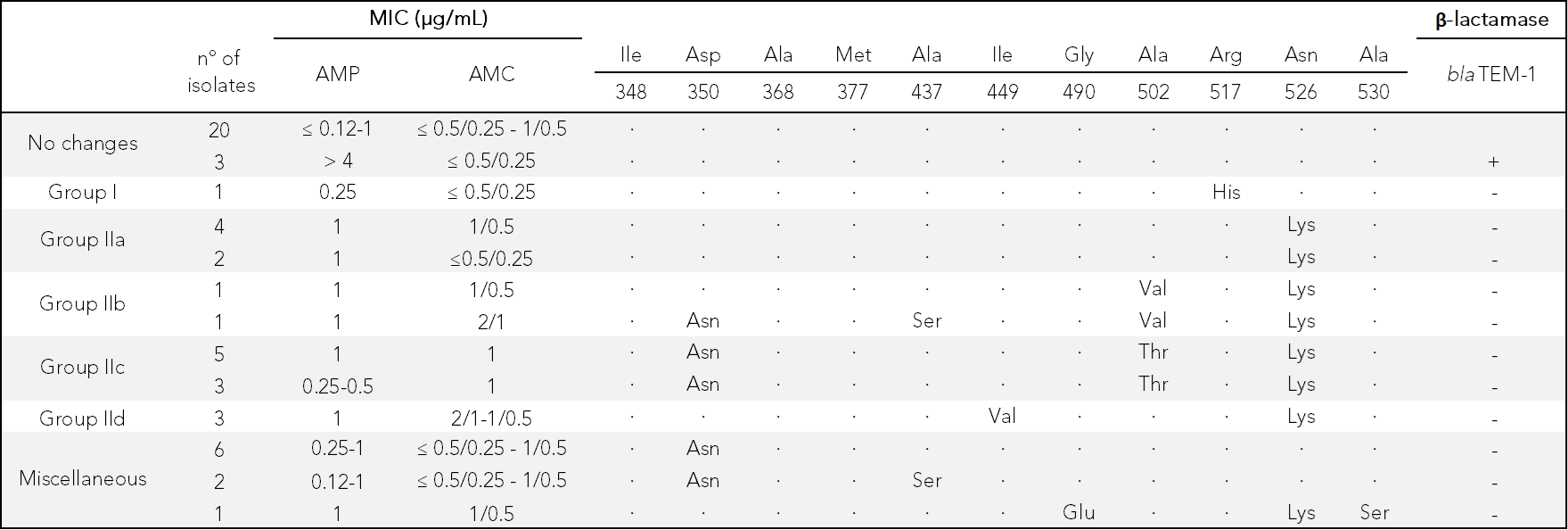

Supplement: Supplementary file 1 — Supplementary Material 1: Supplementary Figure 1. Phylogenetic trees showing different STs found among H. influenzae (A) and P. aeruginosa (B) strains regarding hospitals. Supplementary Table 1. Microorganisms isolated from the 165 non-CF bronchiectasis patients included in the study (2019-2020). *High quality sputum samples were considered for the study. Supplementary Table 2. Cotrimoxazole susceptibility results, tested by microdilution in Haemophilus influenzae strains grown over Mueller–Hinton-Fastidious medium (EUCAST criteria), and associated amino acid modifications in FolA and FolP and mutations in the folA promotor. Supplementary Table 3. Amino acid substitutions identified in the penicillin-binding protein 3 of H. influenzae strains. [file 12931_2026_3553_MOESM1_ESM.zip › Supplementary Table 3.PNG]
